# Leveraging resources and the impact of neglected tropical disease elimination programmes: getting (much) more than what you pay for

**DOI:** 10.1093/inthealth/ihac033

**Published:** 2022-09-21

**Authors:** Iain Jones, Philip Downs

**Affiliations:** Sightsavers, 35 Perrymount Road, Haywards Heath, West Sussex RH16 3BW, UK; Sightsavers, 35 Perrymount Road, Haywards Heath, RH16 3BZ

**Keywords:** aid, development, drug donation, health resources, neglected tropical diseases

## Abstract

Partnerships have been key to significant progress in combating neglected tropical diseases (NTDs). Collaboration between a range of partners, each bringing different skills and resources, helps reach more people in need with greater efficiency. Some partners have contributed in-kind donations, including drugs and volunteer time. When making resource allocation decisions donors need to consider the significant leverage their investment in NTD programmes can have. This commentary estimates the value of the leverage that the Ascend programme achieved. It is clear that funding from UK aid to Ascend delivered (much) more than what they paid for.

Over its short lifespan, the Ascend West and Central Africa programme transformed the lives of millions of people by building upon previous investments to eliminate neglected tropical diseases (NTDs). Working with national governments, local volunteers and global partners, millions of donated treatments were distributed through community- and school-based platforms to protect people from five NTDs. Treating people in this way meant those who were infected were treated, and that infection was less likely to be transmitted among people living in high-risk areas. In the areas targeted by Ascend, this meant that the general health prospects of millions of people improved, and countries were kept on the road to eliminating several NTDs. Making full use of in-kind donations of resources and time also meant that the Ascend programme delivered more for less, enhanced value for money and ensured national programmes left no one behind in the fight against NTDs.

In 2017, the UK government announced they would invest £360 million over 5 y on NTD implementation programmes, protecting hundreds of millions of people as part ‘of an international push to eliminate and eradicate these ailments for good’.^1^ The new investment included the Ascend programme, with a project budget of £220 million that was approved in 2019. It was designed to provide a comprehensive package of interventions to reduce the disability, lost livelihoods and poverty that occur as a result of NTDs^2^ and sought to establish sustainable, integrated NTD programmes in a number of countries.

Since the late 1990s, the UK has supported several NTD programmes tackling disease control and elimination—where feasible—using preventive chemotherapy and transmission control. The Ascend programme protected the significant investment made by the UK over the years and dramatically scaled up UK efforts to eliminate these diseases. The programme was split into two parts–one focusing on South Asia, East and Southern Africa and the other West and Central Africa. The Ascend West and Central Africa programme, with a contract value of £92.7 million^3^, was awarded to Sightsavers in collaboration with the Schistosomiasis Control Initiative Foundation, the Liverpool School of Tropical Medicine and Mott MacDonald. However, because of the UK government's significant cuts to overseas development aid in 2021, only £53.4 million was provided to the programme.

The Ascend business case noted that NTD control and elimination efforts are supported by a unique and highly valued public-private partnership,^4^ with several pharmaceutical companies donating the drugs required for control and elimination efforts. The project document estimated that the full Ascend programme had the potential to deliver treatments ‘worth over £3 billion’.^5^

Many pharmaceutical companies have made public commitments to large-scale drug donation programmes for NTDs, annually donating drugs valued at US${\$}$2–3 billion.^6^ As part of these commitments, GlaxoSmithKline, MSD (trade name of Merck & Co. Inc., Kenilworth, NJ, USA), Eisai, Merck Serono, Pfizer and Johnson & Johnson have generously donated significant quantities of drugs—Albendazole, Ivermectin, Diethylcarbamazine citrate, Praziquantel, Azithromycin and Mebendazole, respectively^7^—to countries knowing that NTD programmes, including Ascend, would ensure efficient and high-quality distribution.

Approximately £17.7 million of the £53.4 million provided by UK aid to Ascend West and Central Africa (hereafter referred to as ‘Ascend’) supported mass drug administration (MDA). This support delivered close to 282 million treatments to almost 117 million people at an average cost per person reached of just £0.15. Sightsavers estimates that during its short lifespan, the Ascend programme leveraged around £607.9 million worth of drugs to support NTD elimination.^a^ This means that every £1 that UK aid provided to Ascend leveraged over £11.4 worth of drugs.^b^ The programme was well on track to contribute its share of the expected £3 billion drug leverage that was noted in the business case. If Ascend had not been closed early, Sightsavers estimates that the planned activities in the final year of the programme would have leveraged an additional £305 million of donated drugs.

It is important to recognise that pharmaceutical companies provide other resources, beyond drug donations, that are difficult to value but are still vital to the goals of the partnership. This includes additional financial and human resources, capacity building and technical advice. The value of these can be considerable and contribute to the delivery of programme outcomes.^8^

Another important factor in the progress made by Ascend was the work of community drug distributors (CDDs) and community health workers (CHWs), the ‘foot soldiers’ of NTD programmes.^9^ CDDs and CHWs volunteer time and effort, often at a considerable cost to themselves, supporting the treatment of people in their communities through MDA. Analysis by Ascend found that CHWs in Ghana can provide up to 45 d for each MDA. It has been reported that CDDs can provide 2.5 working weeks, and up to 11.4 wk in some cases, on NTD programmes,^10^ reducing the time they could be spending on subsistence or income-generating activities.

Community volunteers support many aspects of NTD programmes: social mobilisation and sensitisation around MDA, drug administration door-to-door, educating populations on the prevention and treatment of NTDs, case finding and data collection and census activities, safety monitoring and side effect reporting, as well as the actual MDA itself. The current pandemic has also meant that under the Ascend programme, volunteers were trained to carry out coronavirus disease 2019 (COVID-19) prevention activities. Sightsavers estimates that the value of volunteer time provided by CDDs and CHWs to Ascend was at least £44.7 million.^11^

There were several other in-kind contributions to the Ascend programme that cannot easily be valued. These include resources from endemic national governments; the time taken by teachers on school-based behaviour change interventions; school-based NTD distribution programmes and teacher training; and the skills and capacity of existing health systems. The value of these is likely to be substantial but cannot easily be valued.

Drug donations and volunteer time are in-kind contributions that allow NTD programmes to improve value for money, to deliver at scale and extend the reach of government health services to ‘last mile’ communities.^12^ The in-kind resources provided to Ascend supported faster progress to elimination goals and helped to protect and treat millions of people, transforming more people's lives than would otherwise be possible. UK aid provided an investment of £53.4 million to Ascend; Sightsavers estimates that the programme leveraged at least £653 million worth of additional resources.

Domestic governments, donors and other potential funders of NTD elimination need to recognise that investing in NTD programmes can result in significant leverage of additional resources that allow for greater reach, greater scale and faster progress towards elimination goals. Funders get (much) more than they pay for: leveraged resources help to ensure NTD programmes are a development best-buy and deliver excellent value for money with broad health, social and economic benefits for millions of people living in the poorest regions of the world.^13–15^ These benefits extend beyond the primary scope of eliminating diseases within a set period of time, which is a major public health achievement of its own merit. The additional benefits include strengthening the resilience of primary health systems to manage incident cases of disease morbidity; transition capacities and technologies into longer-term integrated disease surveillance mechanisms; and sustaining preventive health measures that address a wider range of infectious diseases, including COVID-19. Making resource allocation decisions about NTD programmes without considering the resources that can be leveraged, as well as the wider benefits of the investment, would be ill-advised.

## Data Availability

The data underlying this article will be shared on reasonable request to the corresponding author.

## Footnotes

a The estimated value of drug distributed by Ascend is calculated by multiplying the number of treatments for each disease by the number of tablets required per treatment multiplied by the unit cost of each drug.

b The value of disease specific treatment packages is different; final valuation of the donated drugs leveraged is dependent on the co-endemicity of diseases and which diseases are targeted by the programme in any given country.
